# Deep learning-based breast cancer grading and survival analysis on whole-slide histopathology images

**DOI:** 10.1038/s41598-022-19112-9

**Published:** 2022-09-06

**Authors:** Suzanne C. Wetstein, Vincent M. T. de Jong, Nikolas Stathonikos, Mark Opdam, Gwen M. H. E. Dackus, Josien P. W. Pluim, Paul J. van Diest, Mitko Veta

**Affiliations:** 1 Medical Image Analysis Group, Department of Biomedical Engineering, Eindhoven University of Technology, Groene Loper 5, 5612 AE Eindhoven, The Netherlands; 2 Department of Molecular Pathology, Netherlands Cancer Institute, Plesmanlaan 121, 1066 CX Amsterdam, The Netherlands; 3 Department of Pathology, University Medical Center Utrecht, University Utrecht, Utrecht, The Netherlands

**Keywords:** Breast cancer, Biomedical engineering

## Abstract

Breast cancer tumor grade is strongly associated with patient survival. In current clinical practice, pathologists assign tumor grade after visual analysis of tissue specimens. However, different studies show significant inter-observer variation in breast cancer grading. Computer-based breast cancer grading methods have been proposed but only work on specifically selected tissue areas and/or require labor-intensive annotations to be applied to new datasets. In this study, we trained and evaluated a deep learning-based breast cancer grading model that works on whole-slide histopathology images. The model was developed using whole-slide images from 706 young (< 40 years) invasive breast cancer patients with corresponding tumor grade (low/intermediate vs. high), and its constituents nuclear grade, tubule formation and mitotic rate. The performance of the model was evaluated using Cohen’s kappa on an independent test set of 686 patients using annotations by expert pathologists as ground truth. The predicted low/intermediate (*n* = 327) and high (*n* = 359) grade groups were used to perform survival analysis. The deep learning system distinguished low/intermediate versus high tumor grade with a Cohen’s Kappa of 0.59 (80% accuracy) compared to expert pathologists. In subsequent survival analysis the two groups predicted by the system were found to have a significantly different overall survival (OS) and disease/recurrence-free survival (DRFS/RFS) (*p* < 0.05). Univariate Cox hazard regression analysis showed statistically significant hazard ratios (*p* < 0.05). After adjusting for clinicopathologic features and stratifying for molecular subtype the hazard ratios showed a trend but lost statistical significance for all endpoints. In conclusion, we developed a deep learning-based model for automated grading of breast cancer on whole-slide images. The model distinguishes between low/intermediate and high grade tumors and finds a trend in the survival of the two predicted groups.

## Introduction

Breast cancer remains one of the leading causes of death in women^1^. Most breast cancers are invasive ductal carcinomas of no special type (NST), which arise from epithelial cells lining the ducts. In young patients breast cancers tend to be more aggressive and are considered prognostically unfavorable^2,3^. As a result, many breast cancer guidelines recommend (neo)adjuvant systemic treatment for nearly all young patients. However, in some patients locoregional treatment alone could be sufficient and systemic therapy would be overtreatment. Overtreatment can cause serious (age-related) side effects, which could have been prevented and therefore accurate prognostication is necessary to reduce the number of overtreatments^3^.

Histologic tumor grade of NST breast carcinomas is strongly associated with survivorship^4,5^, also in young breast cancer patients^6,7^, and is therefore of great importance in clinical prognostics. In current clinical practice, visual assessment of tissue specimens, using hematoxylin and eosin (H&E) stained slides, is the standard practice for determination of, amongst others, histologic grade. This assessment can be done either under a microscope or on digitized whole-slide images (WSI). The grading system widely used is the Nottingham modification of the Bloom-Richardson system^5,8^ which assesses nuclear atypia, mitotic rate, and degree of tubule formation. Pathologists require extensive training and experience to make these visual assessments. Lack of precision in assessing any of the components leads to subjective grading and poor reproducibility among pathologists^9,10^.

Automated grading may provide a solution by both decreasing pathologist workload and standardizing clinical practice^11,12^. Automated methods to process H&E stained breast histopathology images and identify features associated with grading and survival have been developed before. Most early methods focused on hand-crafted or computer extracted features, such as textural and morphological features derived from statistics of shapes^13–17^. Newer studies that focus on grading and survival prediction by using histopathology images often make use of deep learning^12,18–23^. Deep learning models have been successfully developed for other tasks in breast histopathology^24–33^. In breast cancer grading specifically, most early methods focused on predicting the grading components separately. Methods to capture nuclear atypia^34–38^, tubule formation^15,39,40^ and mitotic count^28,41^ often used labor-intensive nuclei and mitoses annotations as ground truth. These required annotations make it difficult to re-train these methods on newly acquired datasets. More recently, computational pathology methods that work with weak labels on WSI have been developed^42–46^. Through a principle called multiple instance learning (MIL) it is now possible to train an algorithm on an entire WSI with only a global label (e.g. tumor grade).

In this study, we develop a deep learning-based model for automated grading of NST breast cancer on whole-slide images. The model does not require any labor-intensive annotations and distinguishes between low/intermediate and high grade tumors. Training (*n* = 706) and evaluation (*n* = 686) of this model was done on a large dataset (*n* = 1392 patients) derived from the PARADIGM study^2^ with young (age < 40 years) breast cancer patients. Overall survival (OS), distant recurrence free survival (DRFS) and recurrence free survival (RFS) were compared between the predicted low/intermediate and high tumor grade groups.

## Materials and methods

### Patient selection and image acquisition

We use data from the PARADIGM study^2^. This study contains all young (age < 40 years) breast cancer patients without (lymph node) metastases, who had no prior malignancy, did not receive adjuvant systemic treatment according to standard practice at the time of diagnosis, and were diagnosed between 1989 and 2000 in The Netherlands (*n* = 2286). The patients were identified through the Netherlands Cancer Registry. Tumor and normal formalin-fixed paraffin-embedded (FFPE) blocks with corresponding pathology reports were retrieved in collaboration with PALGA: Dutch Pathology Registry^47^. For all patients fresh tumor slides were cut and stained with hematoxylin and eosin (H&E). Estrogen receptor, progesterone receptor, and HER2 were evaluated on fresh stained material^2^. We selected all patients diagnosed with pure ‘invasive ductal carcinoma’ (no special type). Furthermore, we selected patients with complete information on hormone receptor status (estrogen receptor and progesterone receptor), HER2 status, tumor grade, and outcome. For each patient, a pathologist selected one representative H&E WSI. The slides were scanned by the Philips UFS scanner 1.6.1.3 RA (Philips, Amsterdam, The Netherlands) or Nanozoomer XR C12000-21/-22 (Hamamatsu photonics, Hamamatsu, Shizuoka, Japan) at 40× magnification with a resolution of 0.22 µm per pixel. Slides scanned with the Philips scanner were converted to JPEG compressed tiff files. We randomly divided patients into a development set and an independent test set. The model was developed at Eindhoven University of Technology while the clinical information of the test set was stored at the Netherlands Cancer Institute, insuring full independence between training and testing datasets. All experiments presented in this paper were performed in accordance with relevant guidelines and regulations (see also “Ethics approval and consent to participate” section).

### Histopathological assessment

Histologic grading of the WSI into grade 1, 2 or 3 was performed according to the “Nottingham modification of the Bloom-Richardson system”^5,8^. This classification system involves a semi-quantitative evaluation of three morphological components: the degree of nuclear pleomorphism, the percentage of tubular formation and the mitotic count in a defined area. Each component is ascribed a score of 1 to 3 and the final tumor grade is derived from a summation of the three individual components, with grade 1 (low grade) for scores 3–5, grade 2 (intermediate grade) for scores 6–7, and grade 3 (high grade) for scores 8–9.

Tumors were graded by a single pathologist from a team of 16 specialized European breast pathologists each providing the three component grades and the final tumor grade. All grades were converted to low/intermediate (grades 1 and 2) and high grade (grade 3).

### Development of the deep learning model

**Figure 1:**
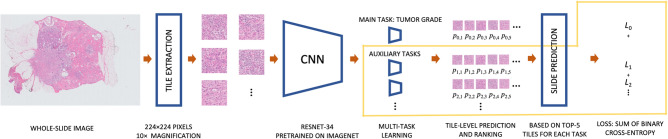
Overview of the deep learning model and training procedure. The components of the model outlined in yellow are used only at training time, this includes the multi-task learning with the auxiliary tasks and the computation of the loss term.

For the development of the deep learning model the patients in the development dataset were randomly assigned to two distinct subsets: training and validation (used for model selection and parameter tuning) datasets. This was separate from our independent test dataset. An overview of the deep learning model and training procedure is presented in Fig. 1.

#### Pre-processing

WSI are generally too large to use as input for a deep learning model, as a result the images needed to be divided into tiles. In order to extract only tiles containing tissue from the WSI we filtered out tiles with mostly white pixels. All tiles were 224 × 224 pixels and sampled at a 10× magnification level. At this resolution both single nuclei as well as larger structures can be observed.

#### Multiple instance learning

A WSI can be seen as a “bag” of tiles and the bag label (i.e. the binary tumor grade) is determined by the prevalence of positive (high grade) instances. This means that a bag is classified as positive (high grade) when it contains at least one positive instance and is classified as negative (low/intermediate grade) when all of the tiles are negative. This assumption is called multiple instance learning (MIL) and is the basis of our model.

The deep learning model was based on the MIL model by Campanella et al.^42^. The backbone of the model has a ResNet-34 architecture^48^ which is pre-trained on ImageNet^49^. In the first step, the inference step, 512 randomly selected tissue tiles per WSI were passed through the network to obtain the probability of being high grade for each tile. For each WSI, the top 5 tiles with the highest probability of being high grade were selected. In the second step, these 5 tiles per WSI were used to train the network. Each of these 5 tiles is assigned the same label as the WSI it belongs to. After the training step, the updated model was used for the next iteration of the inference step. At inference time, prediction for the whole-slide image is made by majority voting for the predictions of the top 5 tiles.

Training was done using stochastic gradient descent optimization with a learning rate of 0.007 and a momentum of 0.9. The mini-batch size was 256 and we applied binary cross-entropy loss. For regularization and model robustness, we used a weight decay of 0.01 and data augmentation was applied to each tile. Data augmentation also helped to overcome the variability of the tissue staining appearance, which is an important hurdle in histopathology image analysis^50^. We used a random combination of 90 degree rotations, horizontal and vertical flips, brightness (up to 0.35), contrast (up to 0.5), hue (up to 0.1) and saturation (up to 0.1). The best performing model (based on Cohen’s Kappa between predicted and actual tumor grade) after 300 iterations was saved.

#### Multi-task learning

In this study, we compared a model trained on tumor grade alone with a multi-task learning (MTL) approach in which we trained models to simultaneously predict tumor grade and other prognostic factors. We trained three deep learning models with different target sets and compared them on our internal validation set before applying the best model to the independent test set. The first deep learning model was trained on tumor grade only, the second model was trained on tumor grade and all three component grades and the third model was trained on tumor grade, component grades and hormone receptor and HER2 status. This was done as we believe that adding component grades and hormone receptor and HER2 status can feed extra discriminatory information to the model. It has been shown that auxiliary tasks can improve generalization as an inductive bias is invoked towards representations that also explain these tasks^51^.

The inference step was similar for each model as the top 5 tiles were selected based only on tumor grade. The model was extended using a hard parameter sharing approach, meaning that all layers were shared for all targets, except for a final densely connected layer that was specific to each task. The unweighted sum of binary cross-entropy losses of all tasks was used as the loss function.

### Statistical analysis

Differences in distributions between the patient characteristics in the development and test dataset were assessed using the Kolmogorov–Smirnov test for continuous variables and Pearson’s chi-squared test with Yates’ continuity correction for categorical variables.

Agreement in grading between pathologists and our model was measured using Cohen’s Kappa and accuracy. Cohen’s Kappa is commonly used for inter-rater agreement and ranges from − 1 to 1, with 1 indicating perfect correlation.

Overall survival was defined as the time from diagnosis until death from any cause. Patients were censored if they were alive at eight years of follow-up. Patients that were lost to follow-up were also censored (*n* = 6). Distant recurrence-free survival was defined as the time from diagnosis until a distant recurrence or death from any cause. Patients who had a second primary tumor before distant recurrence were censored at time of second primary (*n* = 52). Recurrence-free survival was defined as the time from diagnosis until a disease recurrence (local, regional, or distant). Patients who had a second primary tumor before recurrence were censored at time of second primary (*n* = 48). Kaplan–Meier survival analysis was performed using log-rank testing. Hazard ratios were obtained using both univariate and multivariate Cox proportional hazards regression. The multivariate regression was adjusted for tumor size, lymphovascular invasion and locoregional treatment. The proportional hazard assumption was tested using schoenfeld residuals. After stratifying the model for molecular subtype (hormone receptor and HER2 status) none of the variables violated the assumption. All deep learning models were trained using Python version 3.6 and implemented using the PyTorch deep learning framework. All survival analyses were performed using R 4.0 (R Core Team, Vienna, Austria), a two-sided *p* < 0.05 was considered statistically significant.

### Ethics approval and consent to participate

The PARADIGM initiative will use observational data from the NCR and left over archival patient material. All data and material on the young breast cancer patients involved in this study will be used in a coded way. Neither interventions nor active recruitment of study participants will take place within PARADIGM. As a result, the Dutch law on Research Involving Human Subjects Act (WMO) is not applicable. Therefore, the PARADIGM study received a ‘non-WMO’ declaration from the Medical Ethics Committee of the Netherlands Cancer Institute—Antoni van Leeuwenhoek hospital (NKI), waiving individual patient consent, on 31 October 2012 (PTC 12.1489/NBCP project). In addition, approval from the NKI translational research board (TRB) was obtained.

## Results

### Population characteristics

Patient characteristics for our development (*n* = 706) and test (*n* = 686) dataset are summarized in Table 1. The median age for patients in both development and test datasets was 36 years. The distribution of tumor grade and component grades in low/intermediate (grade 1 and 2) versus high grade (grade 3) tumors between the development and test dataset varied by less than 3%. The grade distribution for WSI scanned by the two scanners used in this study were similar (Supplementary Information Table S1). The most common tumor subtype in our dataset was hormone receptor +/HER2−, 56% versus 50% in the development and test dataset respectively. No significant differences were found in the distribution of characteristics between the development and test dataset.

**Table 1:** Patient characteristics of all 1392 women included in our cohort divided in the development dataset (*n* = 706) and test dataset (*n* = 686).

Patient characteristics	Development dataset	Test dataset	*p* value
*n*	706	686	
**Age at biopsy**			1.00
Median years (Interquartile range)	36 (33–38)	36 (33–38)	
**Tumor grade (*n* (%))**			0.25
Grade 1	113 (16)	89 (13)	
Grade 2	244 (35)	238 (35)	
Grade 3	349 (49)	359 (52)	
**Nuclear score (*n* (%))**			0.66
1	20 (3)	16 (2)	
2	335 (47)	315 (46)	
3	349 (49)	354 (52)	
**Tubular score (*n* (%))**			0.62
1	46 (7)	43 (6)	
2	135 (19)	118 (17)	
3	524 (74)	524 (76)	
**Mitoses score (*n* (%))**			0.64
1	249 (35)	230 (34)	
2	170 (24)	161 (23)	
3	286 (41)	295 (43)	
**Subtype (n (%))**			0.06
HR+/HER2−	394 (56)	345 (50)	
HR−/HER2−	182 (26)	218 (32)	
HR+/HER2+	85 (12)	74 (11)	
HR−/HER2+	36 (5)	42 (6)	
**Tumor size (n (%))**			0.22
1A–B	128 (18)	114 (17)	
1C	365 (52)	366 (54)	
2–3	195 (28)	198 (29)	
Missing	18 (3)	8 (1)	
**Lymphovascular invasion (n (%))**			0.81
Absent	587 (83)	566 (83)	
Present	119 (17)	120 (17)	
**Local treatment (n (%))**			0.33
Conserving surgery with radiotherapy	448 (63)	451 (66)	
Mastectomy without radiotherapy	213 (30)	184 (27)	
Other	45 (6)	51 (7)	

*HR* hormone receptor.

#### Model selection on the validation set

Agreement between pathologist and three deep learning models on NST tumor grading is shown in Table 2. The deep learning model trained on tumor grade alone achieved a Cohen’s Kappa score of 0.54 compared to pathologists. The model trained on tumor grade and the three grade components achieved a Cohen’s Kappa score of 0.61 and the model trained on tumor grade, grade components and hormone receptor and HER2 status achieved a Cohen’s Kappa score of 0.58. Although the three models performed comparably, we decided to select the model trained on tumor grade and the three grade components to be applied to the test set. We selected this model as it achieved the highest Kappa score and adding the three grading components to the model adds information that pathologists also use when grading. Further results in this paper are based on this model only.

**Table 2:** Agreement and accuracy of model versus pathologist grading of no special type (NST) tumors.

Model targets	Cohen’s Kappa (SD)	Accuracy (SD)
Tumor grade only	0.54 (± 0.10)	0.77 (± 0.05)
Tumor grade and component grades	0.61 (± 0.09)	0.80 (± 0.05)
Tumor grade, component grades and HR and HER2 status	0.58 (± 0.09)	0.79 (± 0.05)

The results for three models trained on different sets of targets are shown on the validation set (*n* = 142). The standard deviation (SD) was calculated using bootstrapping.

*HR* hormone receptor.

### Final model results on the test set

#### Tumor grading

**Figure 2:**
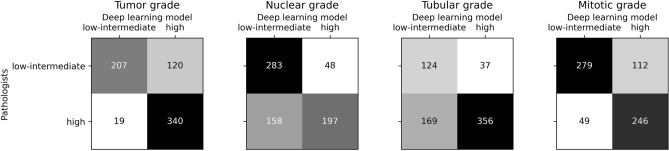
Confusion matrices for no special type (NST) tumor grading and grade components (nuclear, tubular and mitoses scores) between pathologists and the deep learning model. These results are on the test set (n = 686).

Deep learning model versus pathologist agreement for tumor grading and the three grade components is shown in Table 3. Agreement on overall tumor grade between the deep learning model and pathologists was 0.59. The confusion matrices of inter-observer agreement on tumor grading and the grade components can be found in Fig. 2. Interestingly, nuclear and tubular scores showed high numbers of false negatives (23% and 25%), while mitoses score and tumor grading itself showed higher numbers of false positives (16% and 17%).

**Table 3:** Agreement and accuracy of model versus pathologists grading, overall as well as split by nuclear pleomorphism, tubular differentiation and mitotic count.

Target	Cohen’s Kappa (SD)	Accuracy (SD)
Tumor grade	0.59 (± 0.04)	0.80 (± 0.02)
Nuclear score	0.41 (± 0.04)	0.70 (± 0.02)
Tubular score	0.35 (± 0.04)	0.70 (± 0.02)
Mitoses score	0.53 (± 0.04)	0.77 (± 0.02)

Results are shown on the test set (*n* = 686). The standard deviation (SD) was calculated using bootstrapping.

Our model predicts the probability of a tile being high grade cancer for each tile in the WSI. With this information we have created tile-based heatmaps overlayed on the WSI. These heatmaps and the 5 tiles with the highest probability of being high grade are shown in Fig. S1 of the Supplementary Information for 8 WSI.

#### Survival analysis

**Figure 3:**
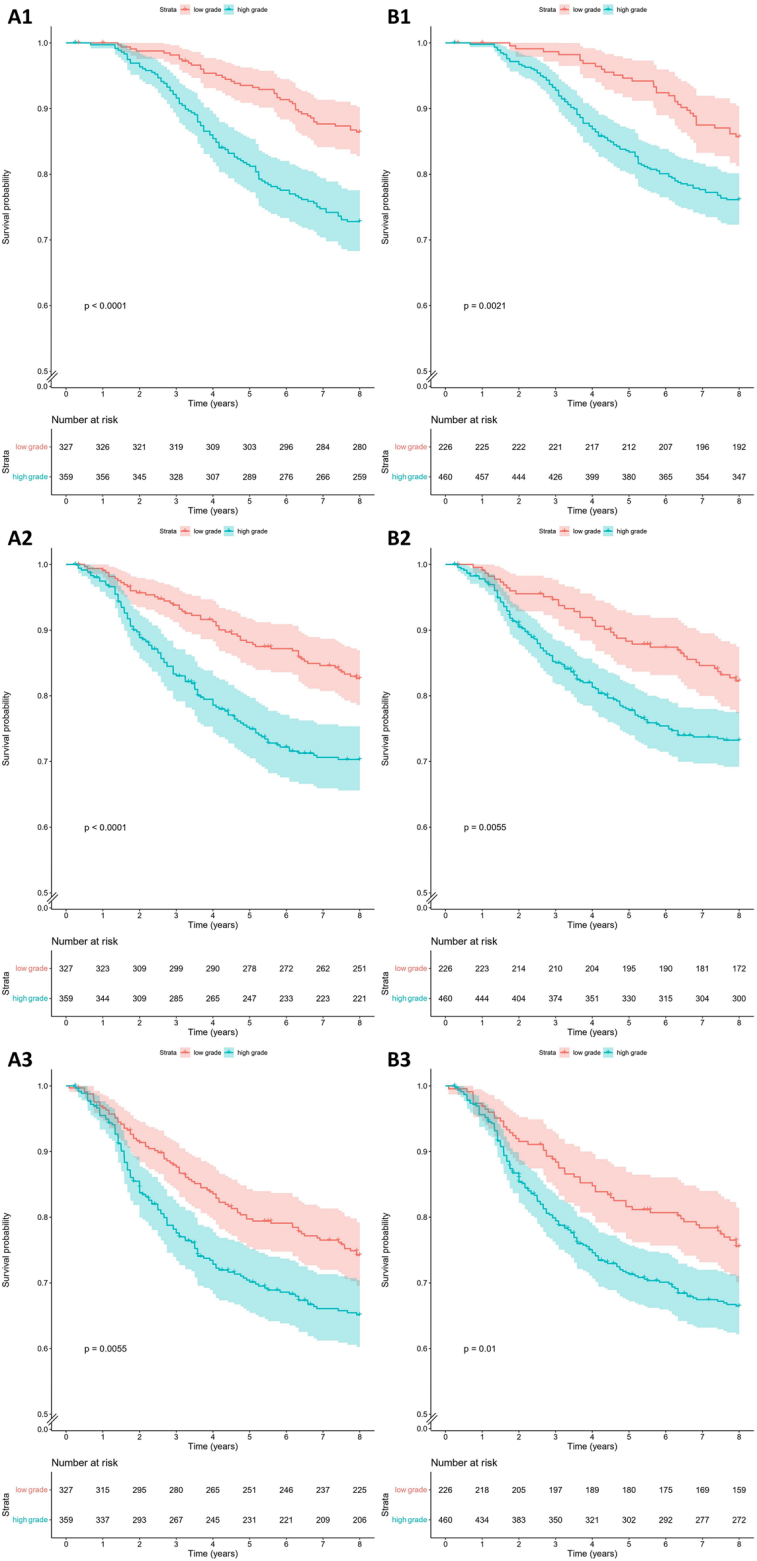
Kaplan–Meier survival curves for young breast cancer patients grouped by low/intermediate versus high grade tumors as assigned by pathologists (**A**) and the deep learning model (**B**) for overall survival (**1**), distant recurrence free survival (**2**) and recurrence free survival (**3**). These results are on the test set (*n* = 686).

Kaplan–Meier curves for patients grouped by low/intermediate versus high grade are shown in Fig. 3. For both pathologists and the deep learning model high grade tumors had a worse prognosis compared to low/intermediate grade tumors for all survival endpoints (OS, DRFS, and RFS). The 8-year survival rates of the low/intermediate and high grade groups as assigned by pathologists and the deep learning model for all survival endpoints are shown in Table 4.

**Table 4:** Eight-year survival rates of low/intermediate and high grade groups as assigned by pathologists and the deep learning model for overall survival (OS), distant recurrence free survival (DRFS) and recurrence free survival (RFS).

Survival endpoint	8-year survival rate (% (95% CI))	
	Pathologists	Deep learning model
**OS**		
Low/intermediate grade	85.7 (81.3–90.4)	86.4 (82.8–90.2)
High grade	76.1 (72.3–80.2)	72.8 (68.3–77.6)
**DRFS**		
Low/intermediate grade	82.7 (78.6–86.9)	82.3 (77.4–87.5)
High grade	70.3 (65.6–75.4)	73.2 (69.2–77.5)
**RFS**		
Low/intermediate grade	74.2 (69.6–79.2)	75.6 (70.1–81.5)
High grade	65.1 (60.3–70.4)	66.5 (62.2–71.1)

Results are shown on the test set (*n* = 686).

We further performed univariable and multivariable Cox regressions for both the deep learning model and pathologist grading. Results of the univariable analysis are shown in Table 5. We found that the deep learning model and pathologist-assigned low/intermediate versus high grade groups were significantly associated with all endpoints (*p* < 0.05). In the multivariable Cox regression, stratified for molecular subtype, the pathologist groups were still significantly associated with OS and DRFS (*p* < 0.05). The deep learning model, however, showed a trend but lost statistical significance (Table 6). Both the pathologist- and model-defined groups were not significantly associated with RFS after adjustment for clinicopathologic features.

**Table 5:** Univariate hazard ratios showing the prognostic value of high versus low/intermediate grade tumors as assessed by pathologists or the deep learning model for different survival endpoints.

Survival endpoint	Pathologists		Deep learning model	
	Hazard ratio (95% CI)	*p* value	Hazard ratio (95% CI)	*p* value
Overall survival	2.23 (1.56–3.19)	< 0.001	1.84 (1.24–2.72)	0.025
Distant recurrence free survival	1.92 (1.38–2.67)	< 0.001	1.66 (1.16–2.39)	0.006
Recurrence free survival	1.49 (1.12–1.97)	0.006	1.50 (1.10–2.05)	0.011

Results are shown on the test set (*n* = 686).

**Table 6:** Multivariate hazard ratios showing the prognostic value of high versus low/intermediate grade tumors as assessed by pathologists or the deep learning model for different survival endpoints.

Survival endpoint	Variables	Pathologists		Deep learning model	
		Hazard ratio (95% CI)	*p* value	Hazard ratio (95% CI)	*p* value
Overall survival	**Tumor grade**				
	Low/intermediate	REF		REF	
	High	1.87 (1.24–2.82)	< 0.01	1.39 (0.89–2.18)	0.15
	**Tumor size**				
	1A–B	REF		REF	
	1C	1.59 (0.91–2.79)	0.11	1.67 (0.95–2.92)	0.07
	2–3	1.54 (0.85–2.80)	0.15	1.67 (0.93–3.03)	0.09
	**Lymphovascular invasion**				
	Absent	REF		REF	
	Present	2.45 (1.68–3.56)	< 0.01	2.61 (1.80–3.78)	< 0.01
	**Local treatment**				
	Conserving surgery with radiotherapy	REF		REF	
	Mastectomy without radiotherapy	0.96 (0.64–1.44)	0.84	1.02 (0.68–1.52)	0.93
	Other	2.23 (1.29–3.85)	< 0.01	2.30 (1.33–3.98)	< 0.01
Distant recurrence free survival	**Tumor grade**				
	Low/intermediate	REF		REF	
	High	1.70 (1.16–2.48)	< 0.01	1.49 (0.99–2.25)	0.06
	**Tumor size**				
	1A–B	REF		REF	
	1C	2.15 (1.19–3.89)	0.01	2.23 (1.23–4.02)	< 0.01
	2–3	2.57 (1.39–4.75)	< 0.01	2.72 (1.48–5.02)	< 0.01
	**Lymphovascular invasion**				
	Absent	REF		REF	
	Present	2.70 (1.91–3.82)	< 0.01	2.87 (2.03–4.04)	< 0.01
	**Local treatment**				
	Conserving surgery with radiotherapy	REF		REF	
	Mastectomy without radiotherapy	1.08 (0.75–1.57)	0.67	1.15 (0.79–1.66)	0.47
	Other	2.24 (1.32–3.81)	< 0.01	2.31 (1.36–3.94)	< 0.01
Recurrence free survival	**Tumor grade**				
	Low/intermediate	REF		REF	
	High	1.30 (0.94–1.80)	0.12	1.37 (0.96–1.96)	0.08
	**Tumor size**				
	1A–B	REF		REF	
	1C	1.64 (1.02–2.61)	0.04	1.65 (1.04–2.63)	0.03
	2–3	1.93 (1.18–3.17)	< 0.01	1.97 (1.21–3.21)	< 0.01
	**Lymphovascular invasion**				
	Absent	REF		REF	
	Present	2.70 (1.98–3.68)	< 0.01	2.75 (2.02–3.73)	< 0.01
	**Local treatment**				
	Conserving surgery with radiotherapy	REF		REF	
	Mastectomy without radiotherapy	1.00 (0.72–1.39)	1.00	1.02 (0.74–1.42)	0.88
	Other	1.72 (1.05–2.82)	0.03	1.74 (1.06–2.85)	0.03

This model was stratified by molecular subtype. Results are shown on the test set (*n* = 686).

## Discussion

In this study, we trained a deep learning-based breast cancer grading model that works on entire WSI. The model distinguishes between low/intermediate and high grade tumors and also predicts nuclear, mitotic and tubular grade. It was developed using a dataset of H&E WSI from young breast cancer patients, a group for whom current breast cancer prognostication tools are not adequately validated^2^. The deep learning-based breast cancer grading model was able to pick-up a non-significant trend in outcome between the two predicted grade groups (low/intermediate vs. high).

The inter-observer agreement between the model and pathologists, as measured by Cohen’s Kappa, was 0.59 (accuracy 80%) for distinguishing between low/intermediate and high tumor grade on the test set, which is considered moderate. Tumors with pathologist-defined intermediate grade were more likely to be misclassified as high grade tumors by the model than pathologist-defined low grade tumors (results not shown). The agreement of the trained model with the grading of pathologists was slightly lower than the agreement found between two breast pathologists for the same task on a different dataset (kappa 0.78, accuracy 89%)^23^.

Using our model we also evaluated grading components separately (nuclear, tubular, and mitosis scores). We found Kappa scores for model versus pathologist agreement between low/intermediate and high grade of 0.41, 0.35, and 0.53, for the nuclear, tubular, and mitotic component, respectively. In contrast to our results, previous studies among pathologists found scoring tubule formation was more reproducible than scoring either nuclear pleomorphism or mitotic count^52–55^. We assume, that the low reproducibility of tubular grade in our study is due to the MIL framework not being suited to scoring this component. The model predicts grades for each component based on the top 5 tiles extracted from the WSI that are predicted to have the highest overall tumor grade. Nuclear pleomorphism and mitotic count can easily be scored on these top 5 tiles. However, since tubular formation is scored as a percentage of the entire tumor area, the model prediction based on only 5 tiles seems not to be able to fulfill this task.

Our method is based on the MIL methodology developed by Campanella et al.^42^. The method was originally tested on tasks and datasets that are substantially different than our tasks (prostate, skin and lymph node status) so a direct comparison of the performance is not adequate. We used a version of the method that works with 10× magnification, which offers a good compromise between details of the morphology that are visible and computational speed. We selected this magnification offers a good balance between each tile containing tissue architecture information and sufficient details of the morphology of the individual nuclei.

Since the dataset that we used for developing our model contained additional global labels, such as the different components of the grade and HR and HER2 status we investigated a multi-task learning approach. Such models can learn better (based on multiple tasks) representation of the image data and should always be investigated if such auxiliary tasks are available. The MTL model that in addition to the tumor grade also predicts the grade components resulted in the highest Cohen’s kappa and accuracy on the validation set and was used in all subsequent analyses on the test set.

Despite successfully performing deep learning models for breast histopathology tasks^23–32^, using deep learning is discouraged due to its lack of interpretability^56,57^. To better interpret the results of the model we created heatmaps (at the tile level) and extracted the top 5 tiles for 8 WSI shown in Supplementary Information Fig. S1. These heatmaps make it possible for pathologists to see which regions the model considers when making decisions. Supplementary Information Fig. S1 shows that, although no tumor annotations were used in this study, the model clearly focuses on the tumor area when grading. Furthermore, the top 5 selected tiles for high grade tumors often contain high nuclear scores and high mitotic activity. Such correlations between the morphological appearance of the tissue (such as the size, texture and type of nuclei and their organization) of the selected tiles and the predicted class label of the model should be further investigated in future work with the goal of improved interpretability of the model. Furthermore, if used in clinical practice, interpretability tools can be used by clinicians for rejecting spurious predictions (e.g. if the selected tiles are clearly located in irrelevant areas such as fat tissue). However, the precise mechanism of this should be further investigated in future work.

When assessing potential biomarkers, both robustness and validity are essential to confirm clinical applicability. Deep learning models are robust in the sense that the exact same slide, per definition, will always produce the same grade. However, this may not hold if the slide was scanned on a different scanner or was otherwise changed (e.g. due to faded staining). Our results were validated by analysis on a large (*n* = 686) independent, but similar sample set. This test set included samples from different institutes but all slides were, stained and scanned at the same institute. However, FFPE was made in different institutes.

Kaplan–Meier survival analysis showed a significant difference (*p* < 0.05) in OS, DRFS and RFS for both pathologist- and model-defined low/intermediate versus high grade groups on our test set. In all cases, the difference between the two groups is slightly larger for pathologist-defined groups. Univariable hazard ratios for OS, DRFS and RFS Cox regression were statistically significant for both the model and pathologists (*p* < 0.05). After adjusting for clinicopathologic features and stratifying for molecular subtype the hazard ratios were still significant for pathologists (OS and DRFS) but not for the deep learning-based model, which showed a non-significant trend. Currently pathologists are better at predicting patient outcomes than the deep learning model. However, the trend that the deep learning model shows holds promise for the future.

Our model offers some important advances over previously described breast cancer grading models with comparable performance (e.g.^23^). Firstly, the fact that it was developed using a weakly supervised learning approach without time-consuming detailed annotations. This means that the model can be directly applied to a WSI (no specific manual area selection needed). Secondly, our work distinguishes itself because it was developed on a large dataset of WSI from young breast cancer patients. The trained model shows promise for further development and validation of prognostic deep learning-based tools in this group. Thirdly, the model we have trained is interpretable for pathologists because it can show which regions in a WSI it uses for its assessment of tumor grade. This is an important step for the adoption of these models as a second reader in daily clinical practice.

Our work should be viewed in light of some limitations. Firstly, our model only discerns low/intermediate from high grade tumors and cannot discern between low (grade 1) and intermediate (grade 2) tumors. Grade 1 tumors have a more favourable clinical course and this highly relevant clinical information is lost when using the model. Secondly, our analysis lacks several control groups. The first control group would regard WSI scanning. Our study includes two different scanners but each WSI was only scanned by one scanner. Re-scanning slides on both platforms could help us compare robustness and validity of grade estimates. The second control group could be created by having multiple pathologists grade each WSI. Breast cancer grading remains difficult and could result in moderate reproducibility of tumor grade^10,52^. In this study, the WSI were each graded by a single pathologist from a group of very experienced breast pathologists. Insufficient consistency between tumor grades in the training dataset can make it harder for the model to learn correct patterns for the different grades. To create a model for objective breast cancer grading, objective ground truth annotations need to exist. In this dataset higher quality grades for training and testing could be achieved by using consensus grades of multiple pathologists for each WSI. Thirdly, due to the skewed distribution of breast cancer molecular subtypes in young patients we cannot be sure that the model will perform similarly for older women with breast cancer. Finally, it should be noted that deep learning models are made to function in developed countries with state-of-the-art laboratories that have all needed hardware and software in place. These techniques are, therefore, not available to everyone, everywhere.

Another way to work with more objective targets would be to train our model on survival endpoints directly. Future work can include the investigation of a fully automated approach for breast cancer prognostication. Our model could directly be used to predict binary 5 or 10-year survival on WSI. Furthermore, it could be adapted to work with Cox proportional hazards like several groups working on other pathology tissues have done^58–60^. Prior work on deep learning for breast cancer prognostication specifically has used existing clinically validated risk models (such as ROR-PT score^23^) as a proxy for long term outcome, but none have predicted survival directly.

In conclusion, we have trained a deep learning-based breast cancer grading model. The model can be applied to entire WSI directly, does not need time-consuming detailed annotations and was validated on a large dataset of young breast cancer patients. It distinguishes between low/intermediate and high grade tumors and shows a non-significant trend for the prediction of patient outcome. This model is a first step, and is not yet ready to be applied in clinical practice as at this point in time pathologists still outperform the model in predicting survival. Furthermore, any potential sources of bias in the predictions should be thoroughly investigated before applying such a model in practice. In the future deep learning models may be used to aid pathologists in making even more robust assessment of breast cancer tumor grade, especially in cases with less straight forward morphology. In addition, future work may include the investigation of a fully automated approach for breast cancer prognostication using deep learning-based models like ours to directly predict patient outcome on WSI.

## Supplementary Information

Supplementary Information.

## Data Availability

Subject clinical data, whole slide images, and pathological reviews that support the findings of this study are not publicly available. The survival data that support the findings of this study are available from the Netherlands Cancer Registry, hosted by the Netherlands Comprehensive Cancer Centre (IKNL) but restrictions apply to the availability of these data, which were used under license for the current study. Data are available from the authors upon reasonable request and with permission of The Netherlands Comprehensive Cancer Centre (IKNL).
