# Supplementary material for: Deep learning-based breast cancer grading and survival analysis on whole-slide histopathology images
Source: Sci Rep. 2022 Sep 6;12:15102. doi: 10.1038/s41598-022-19112-9 (PMC9448798; doi:10.1038/s41598-022-19112-9)
Supplement: Supplementary file 1 — Supplementary Information. [file 41598_2022_19112_MOESM1_ESM.docx]

**Supplementary Information**

**
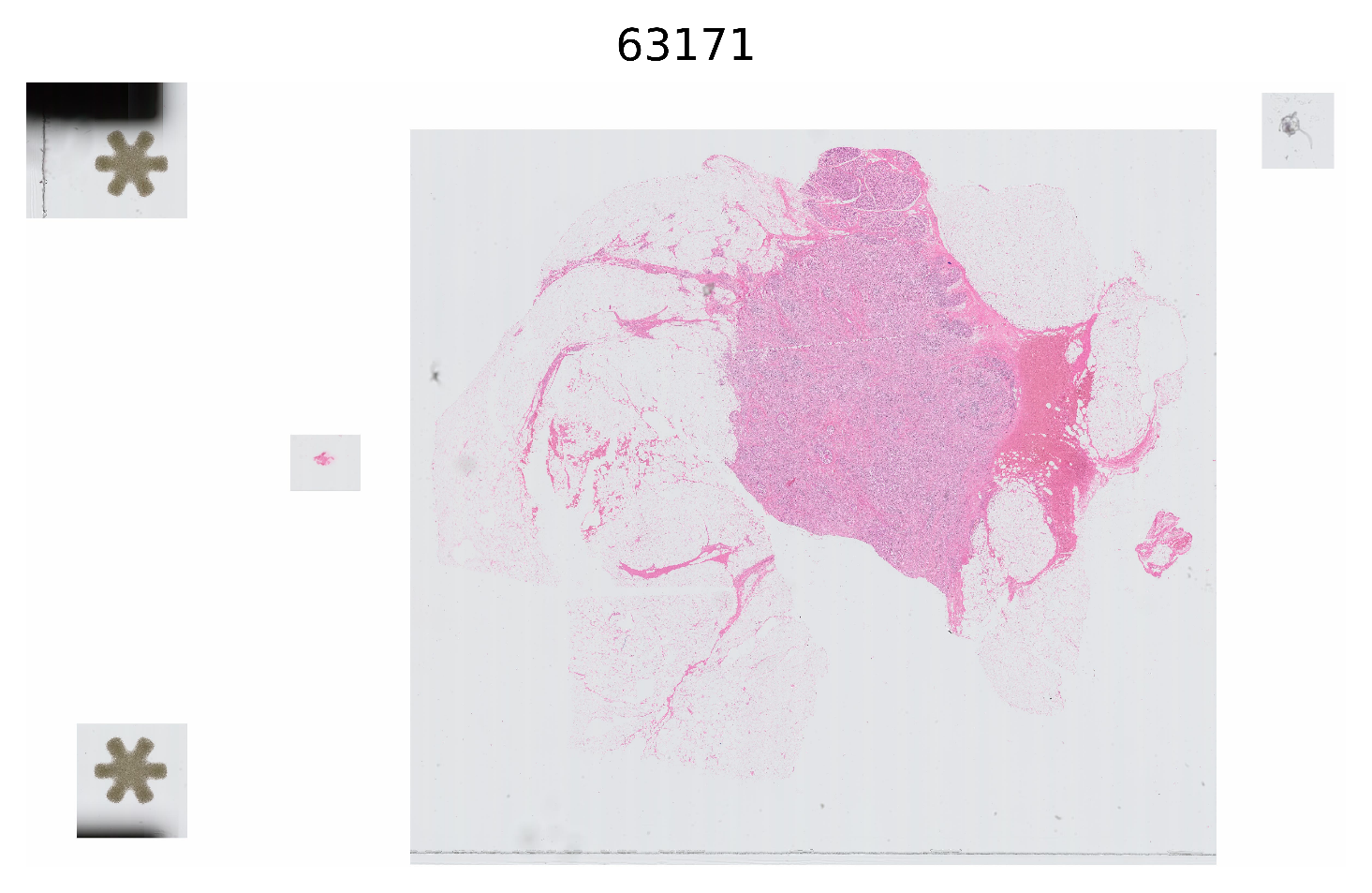

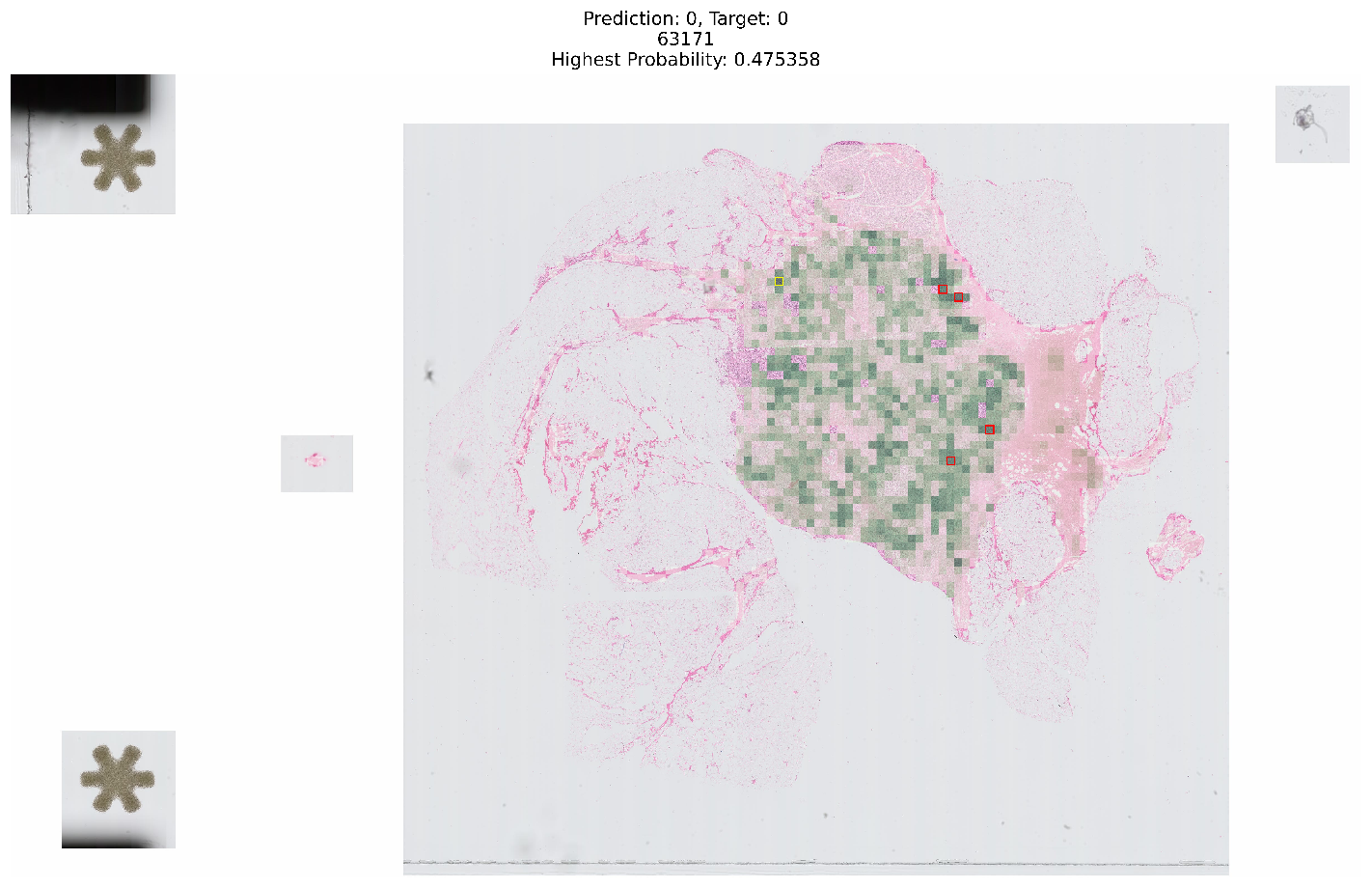
**

**
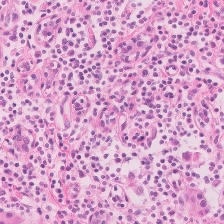

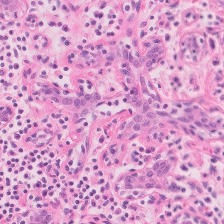

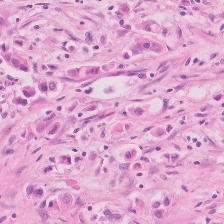

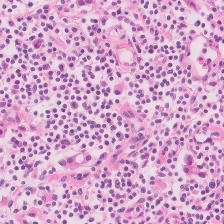

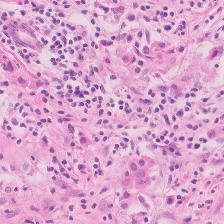
**

(a) Heatmap and top 5 tiles for a correctly classified low-intermediate grade cancer.


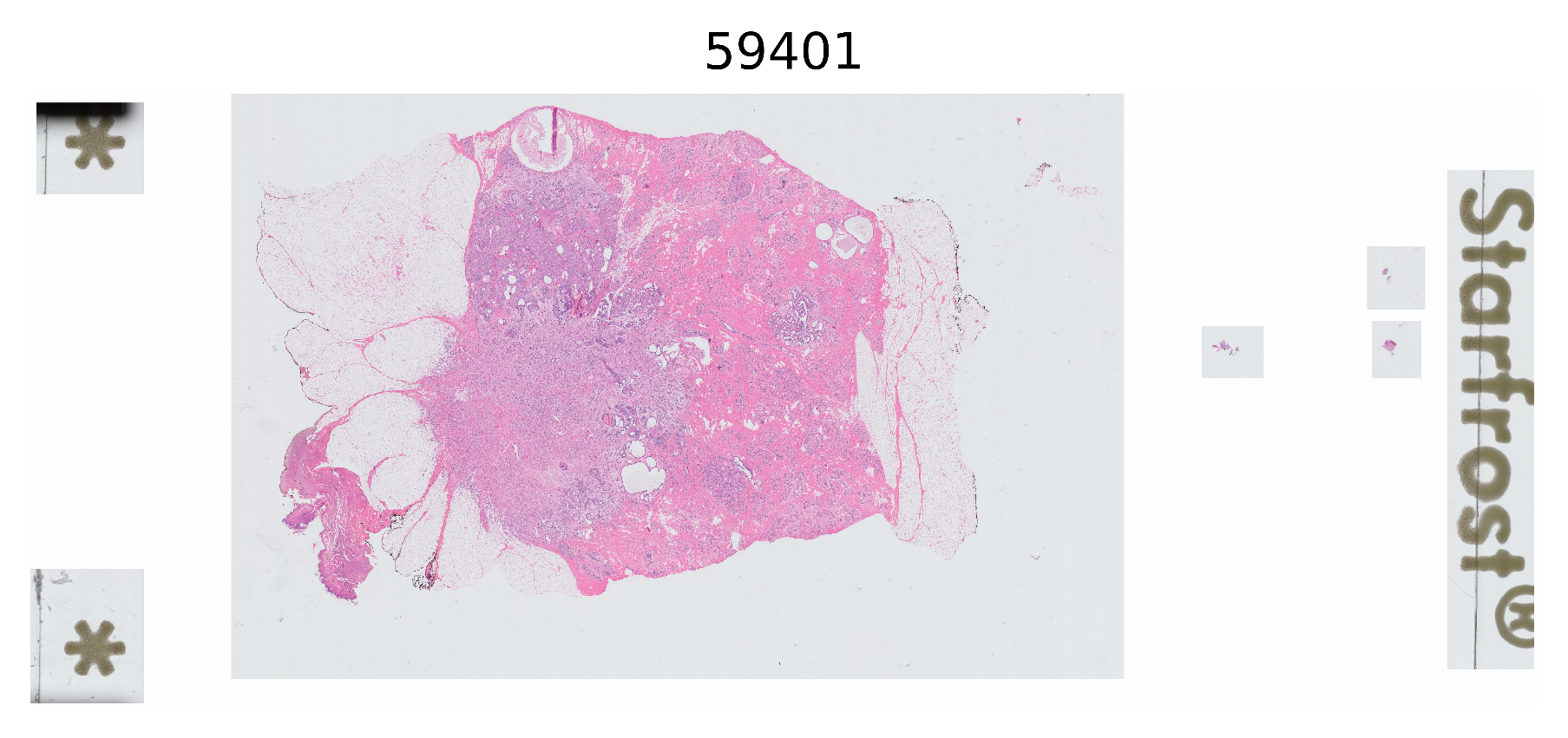

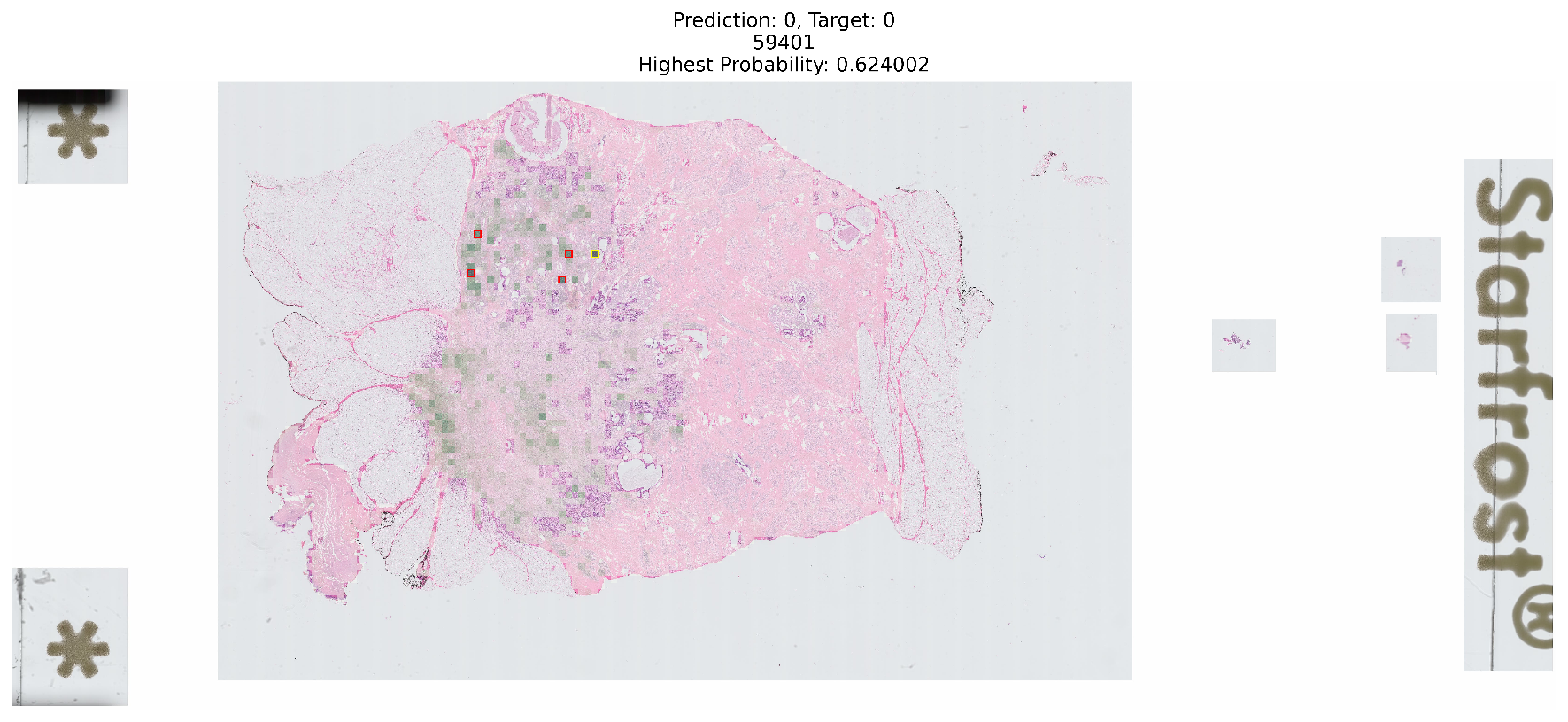


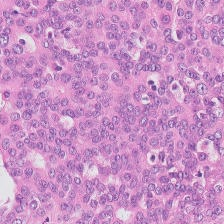

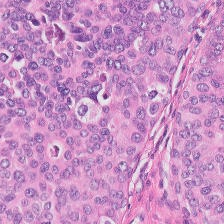

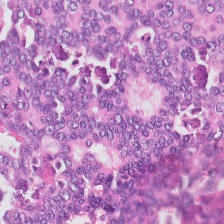

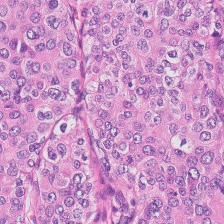

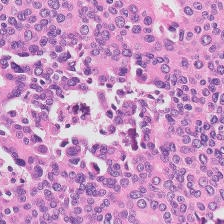


(b) Heatmap and top 5 tiles for a correctly classified low-intermediate grade cancer.


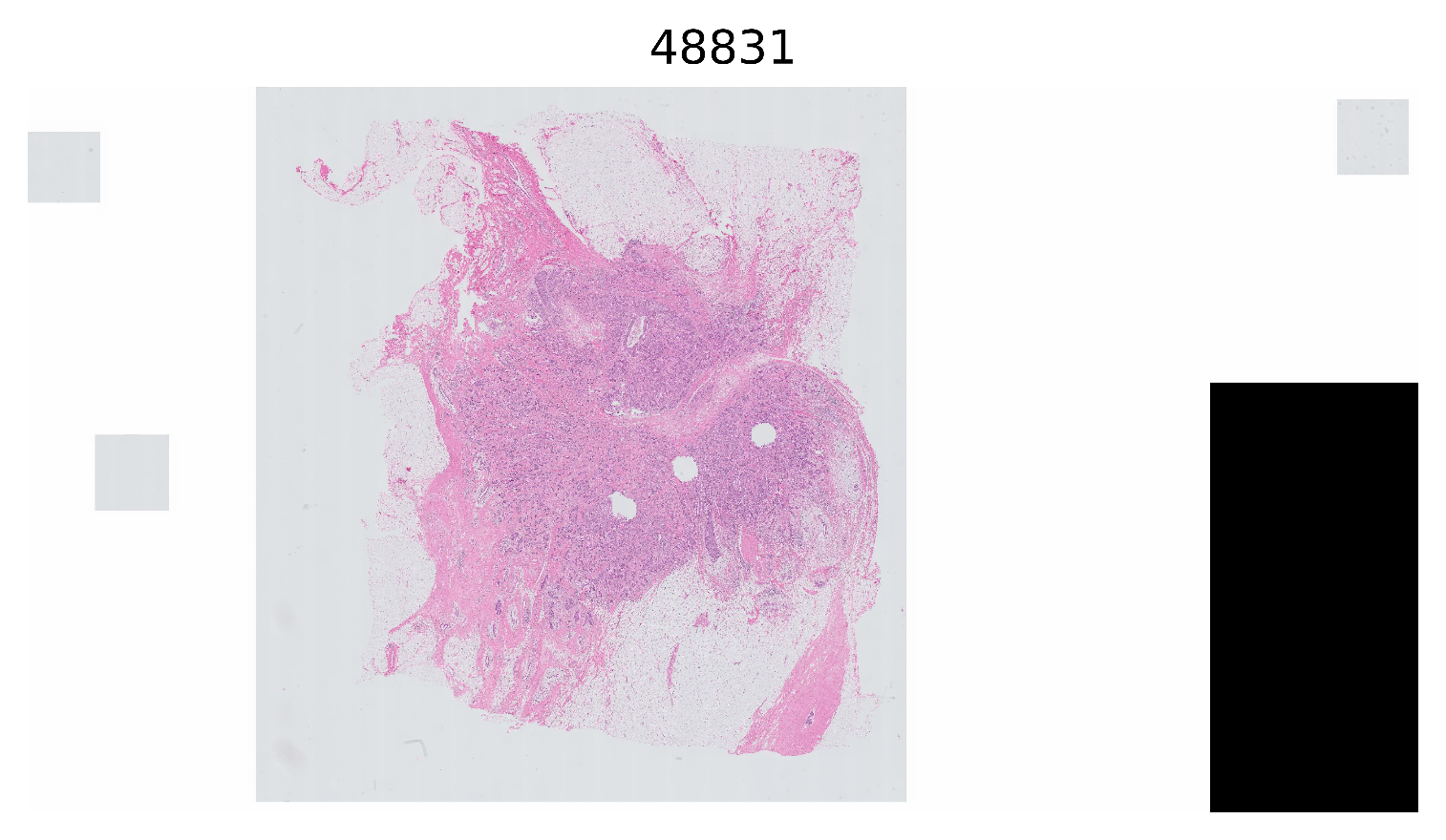

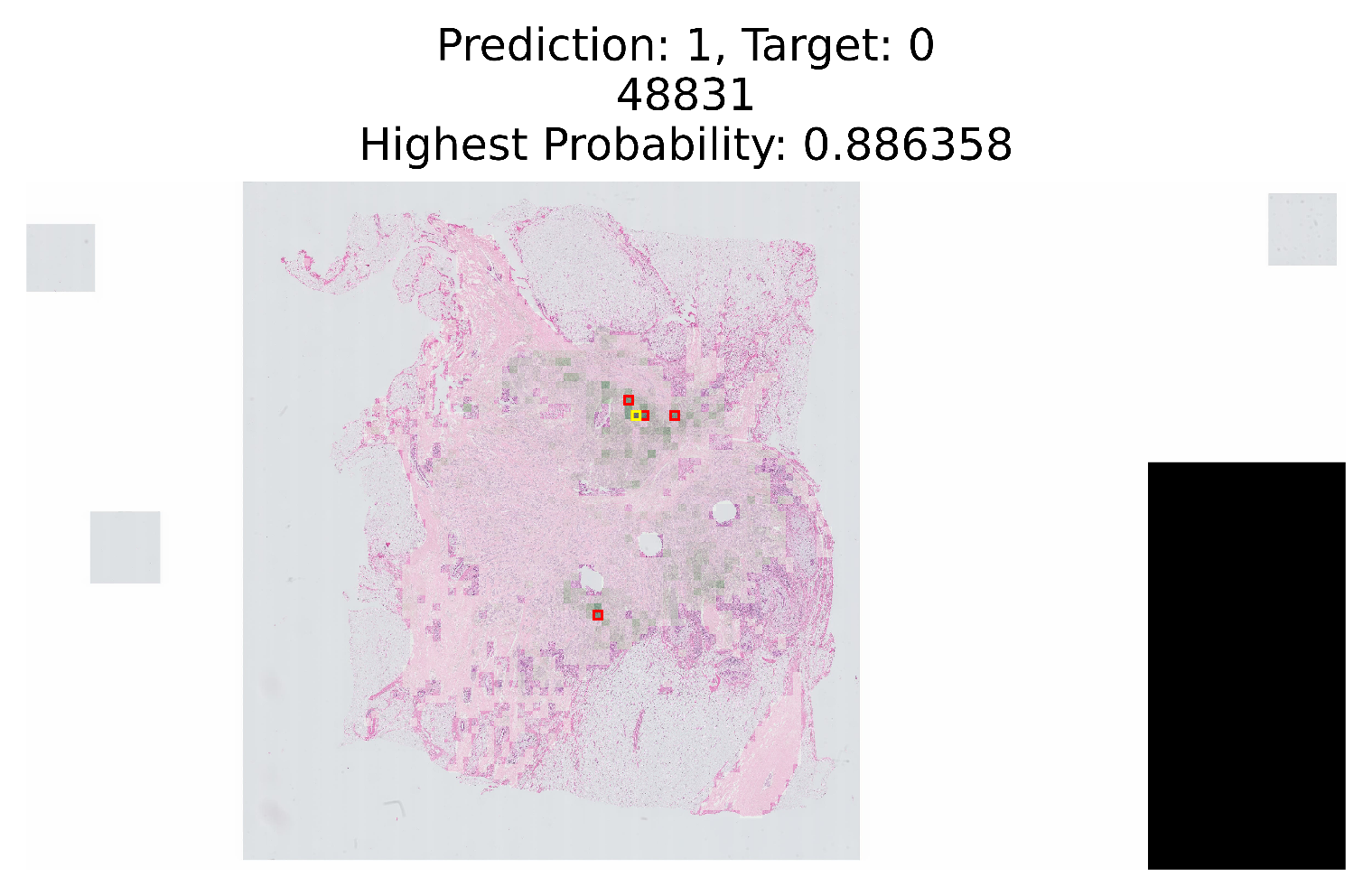


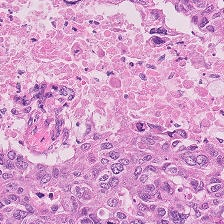

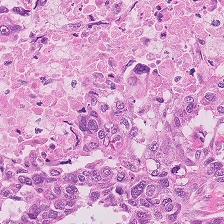

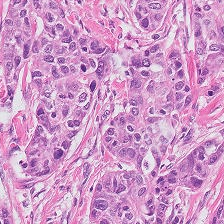

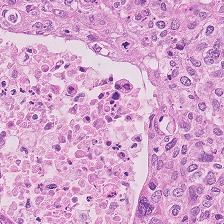

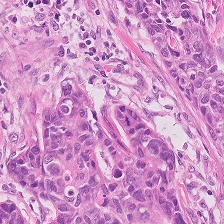


(c) Heatmap and top 5 tiles for a low-intermediate grade cancer classified as high grade.


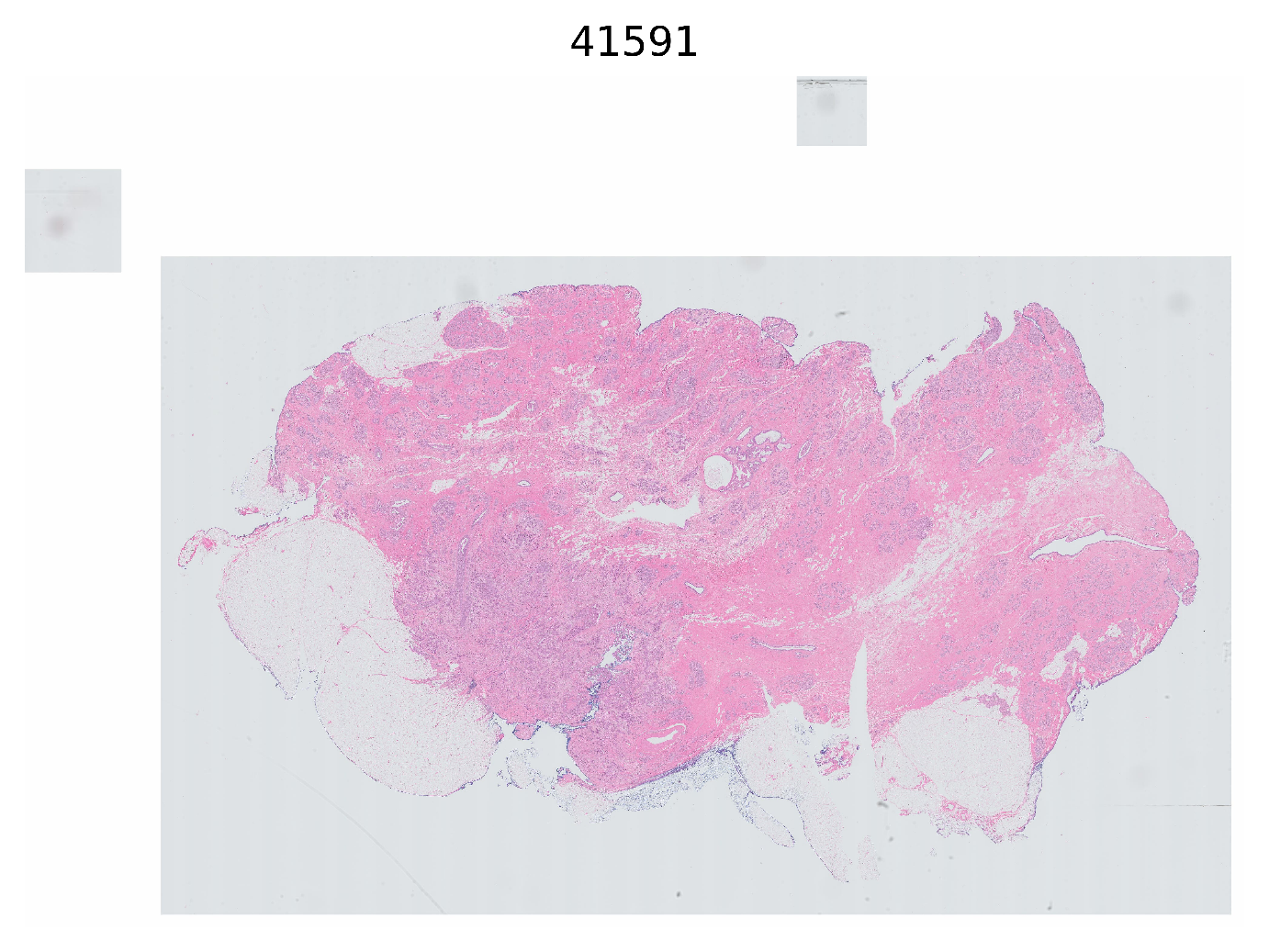


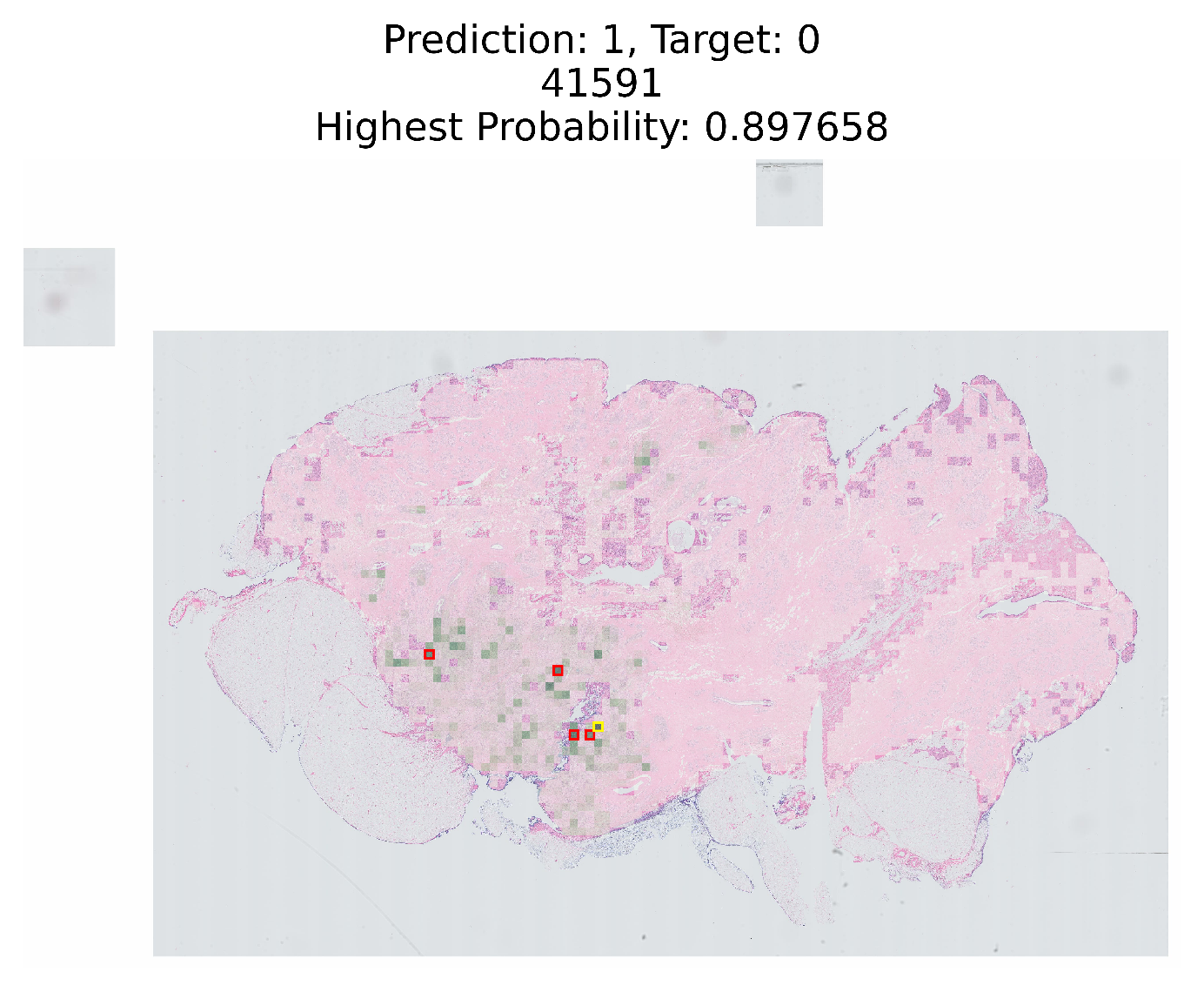


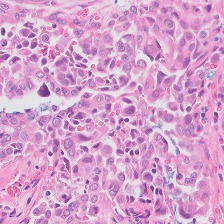

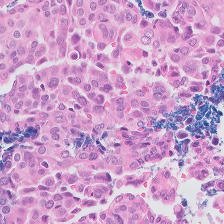

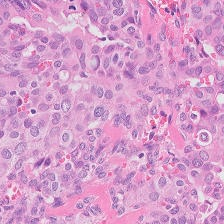

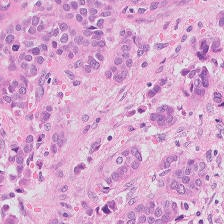

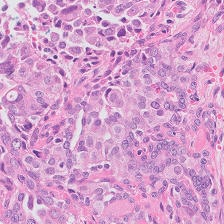


(d) Heatmap and top 5 tiles for a low-intermediate grade cancer classified as high grade.


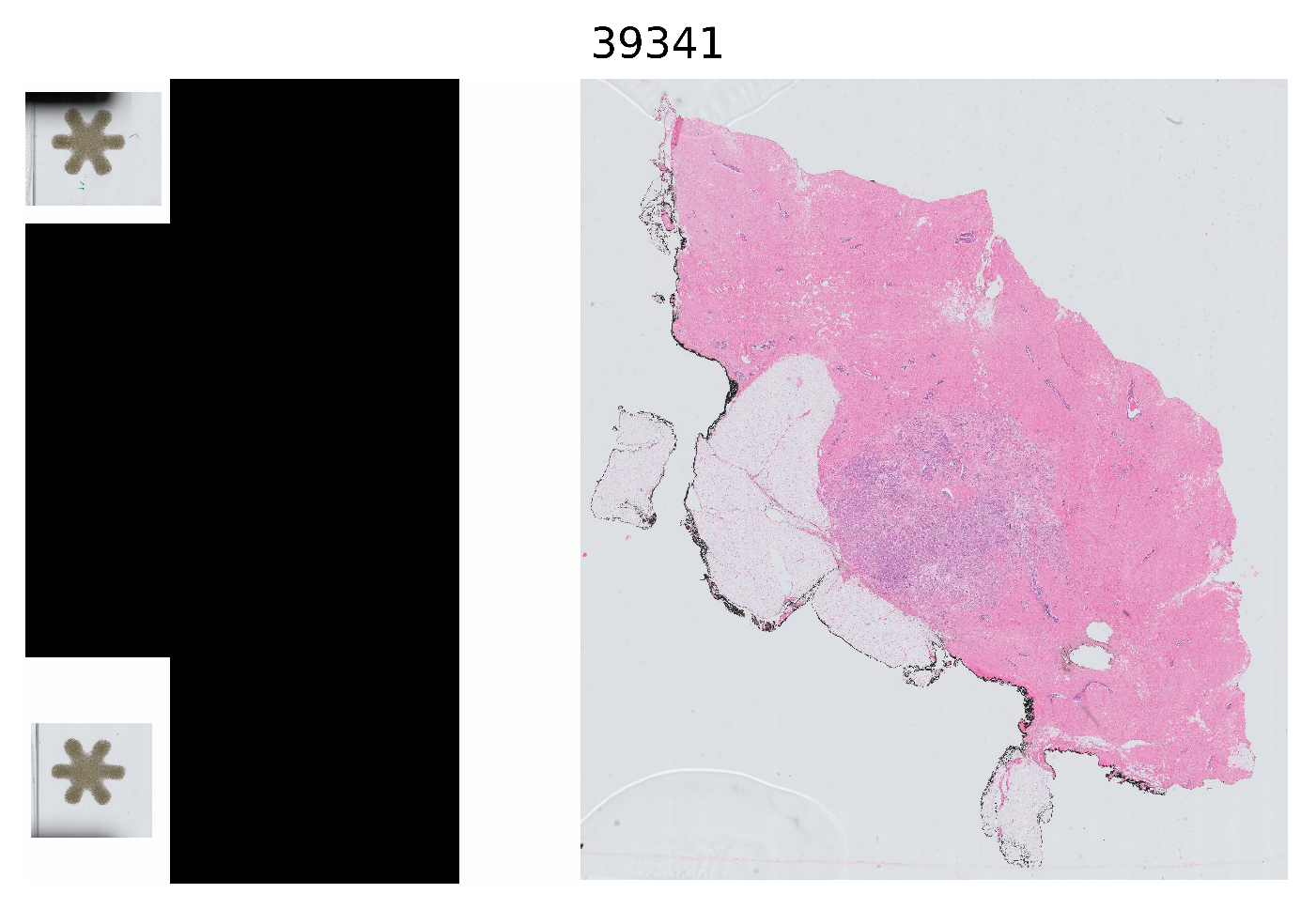

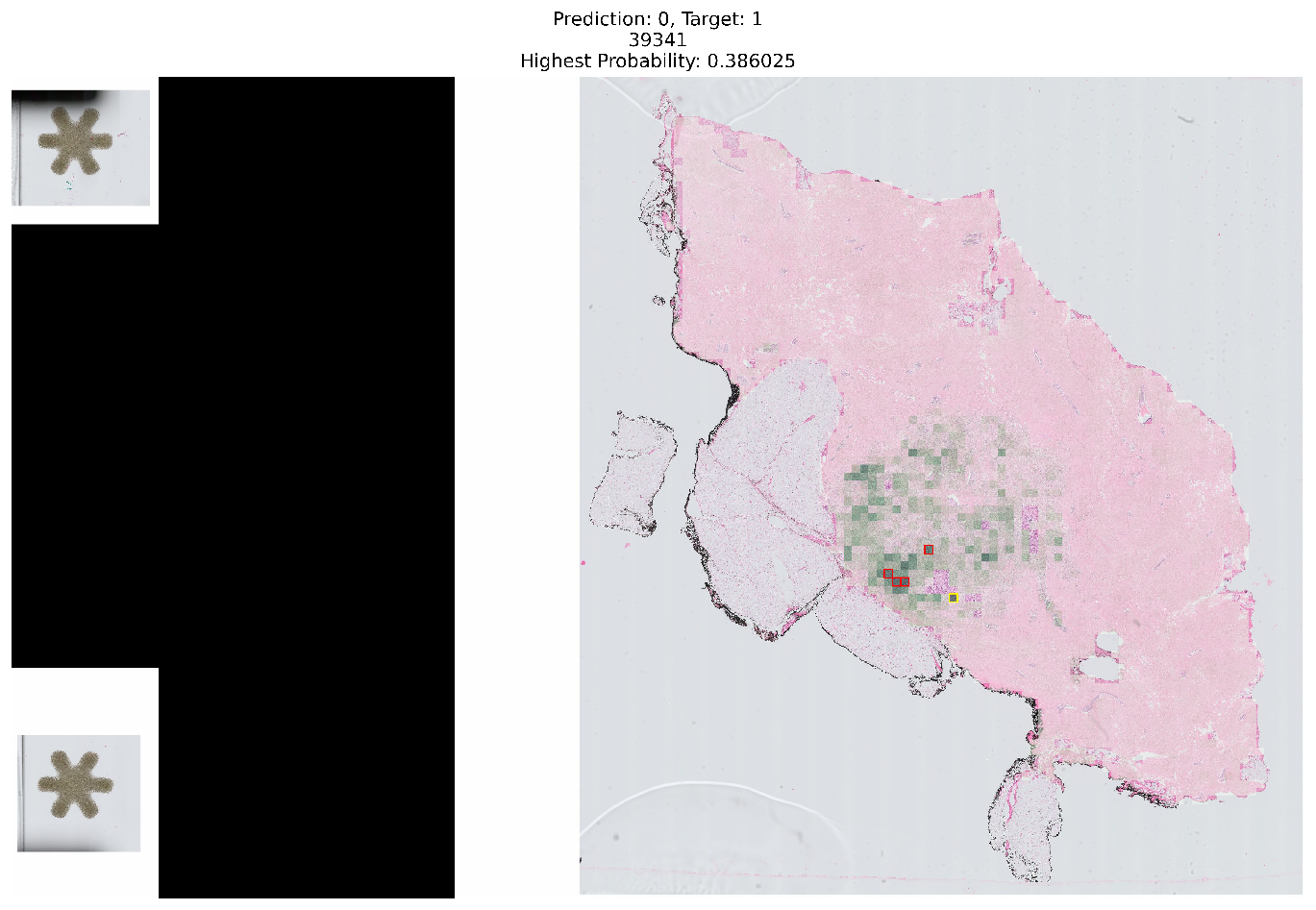


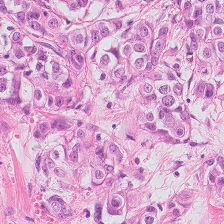

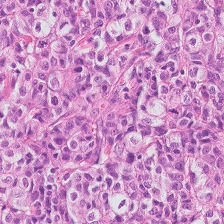

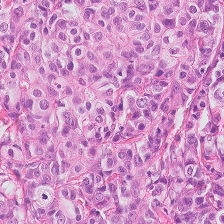

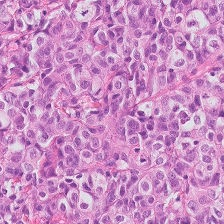

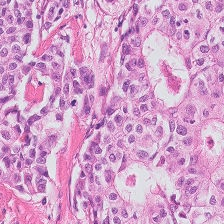


(e) Heatmap and top 5 tiles for a high grade cancer classified as low-intermediate grade.


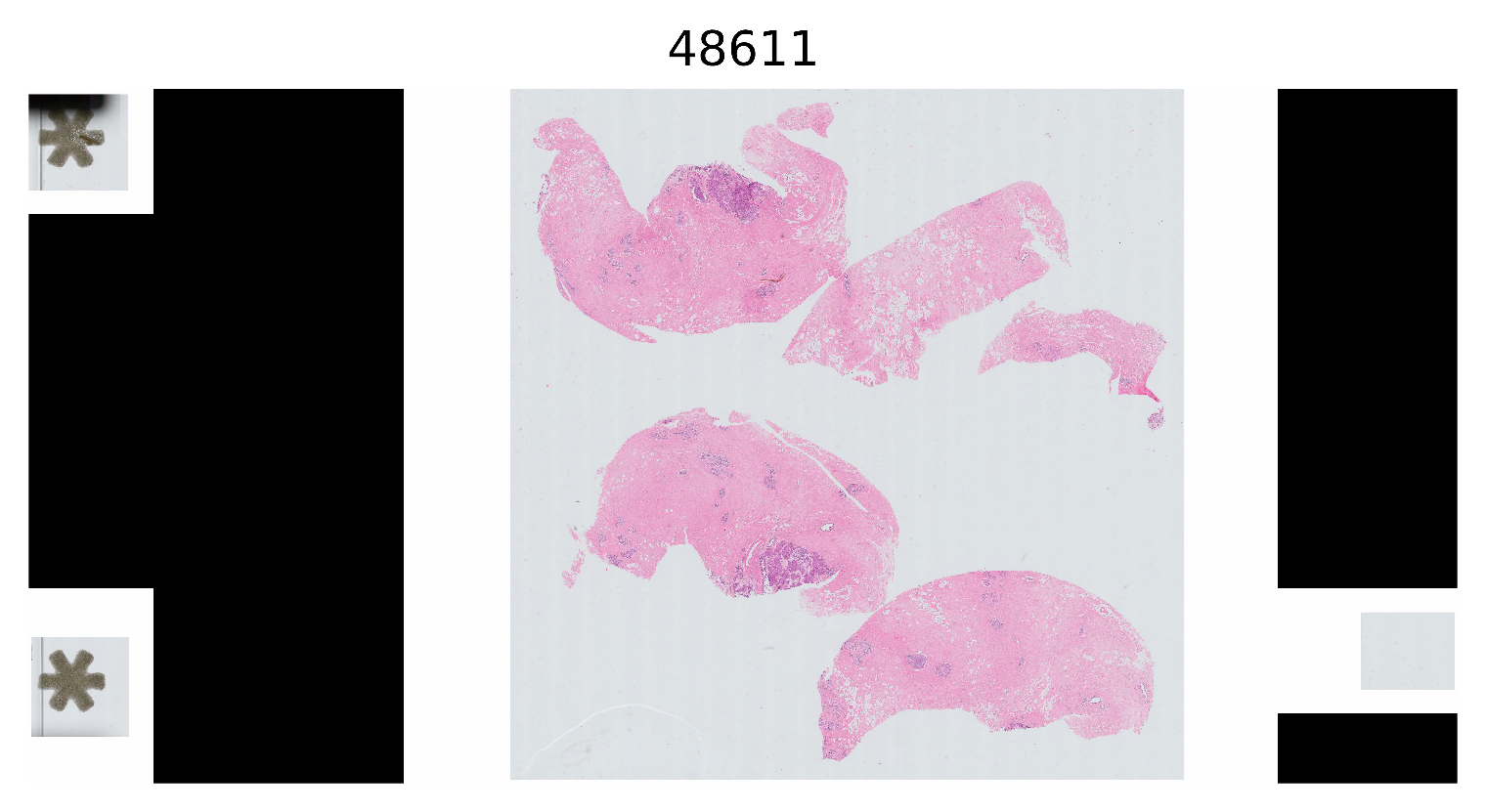

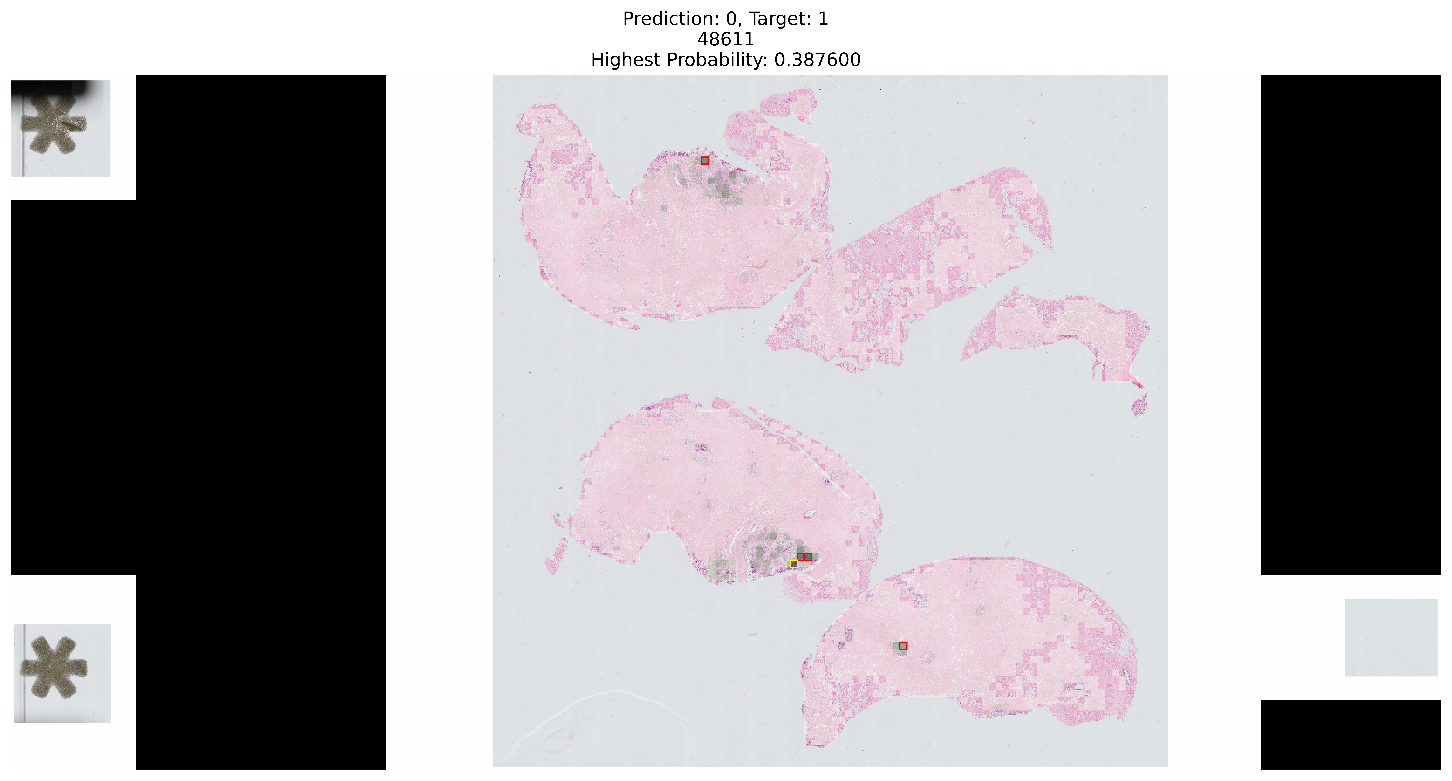


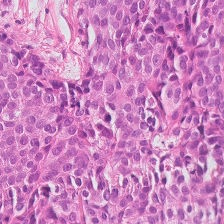

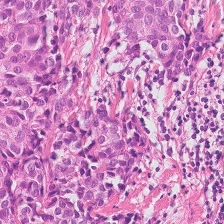

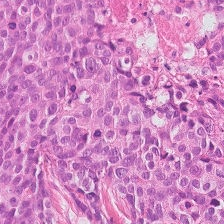

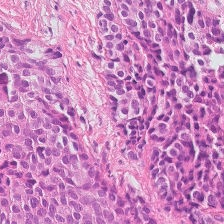

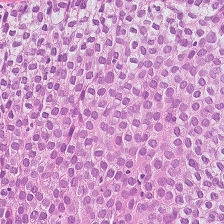


(f) Heatmap and top 5 tiles for a high grade cancer classified as low-intermediate grade.


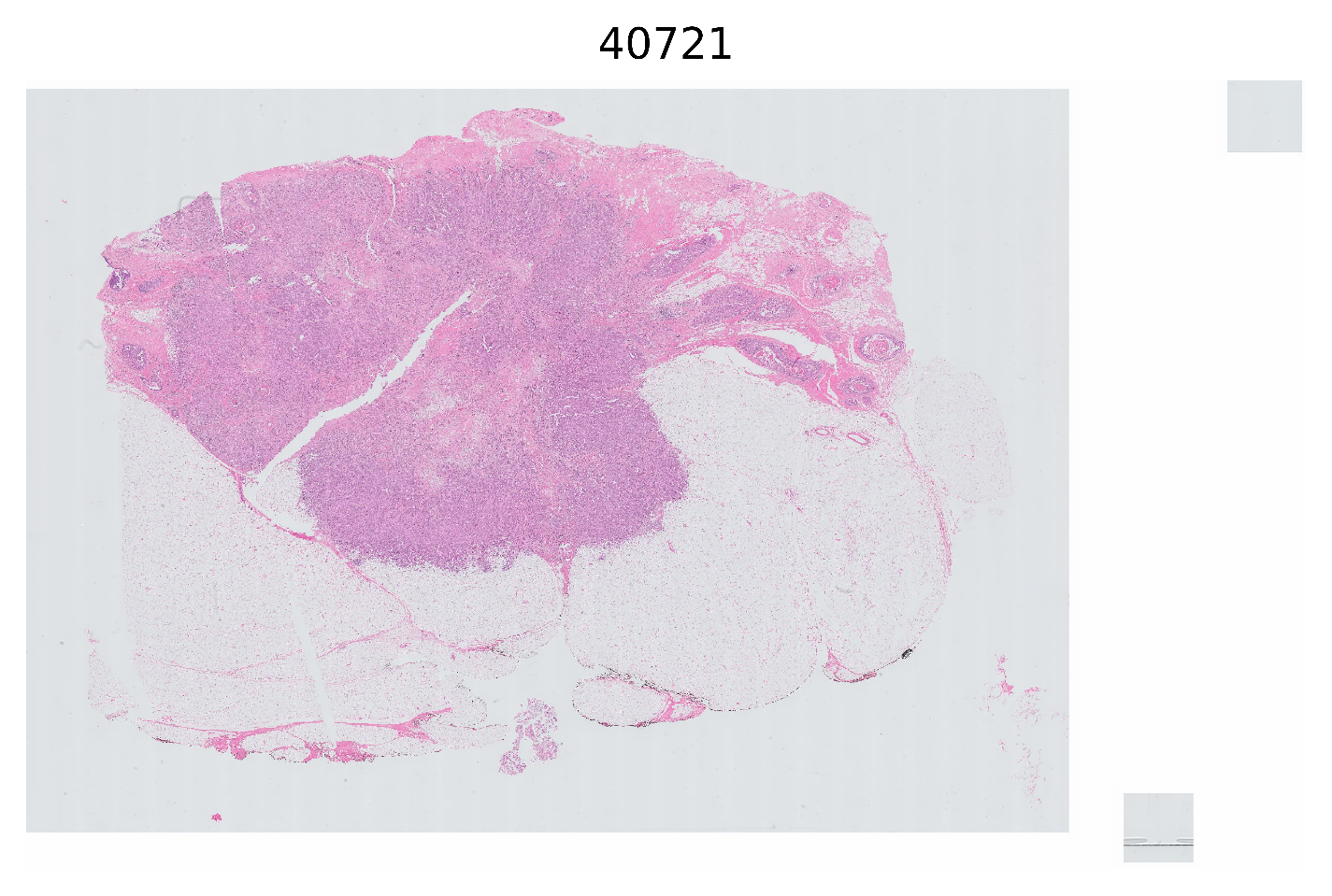

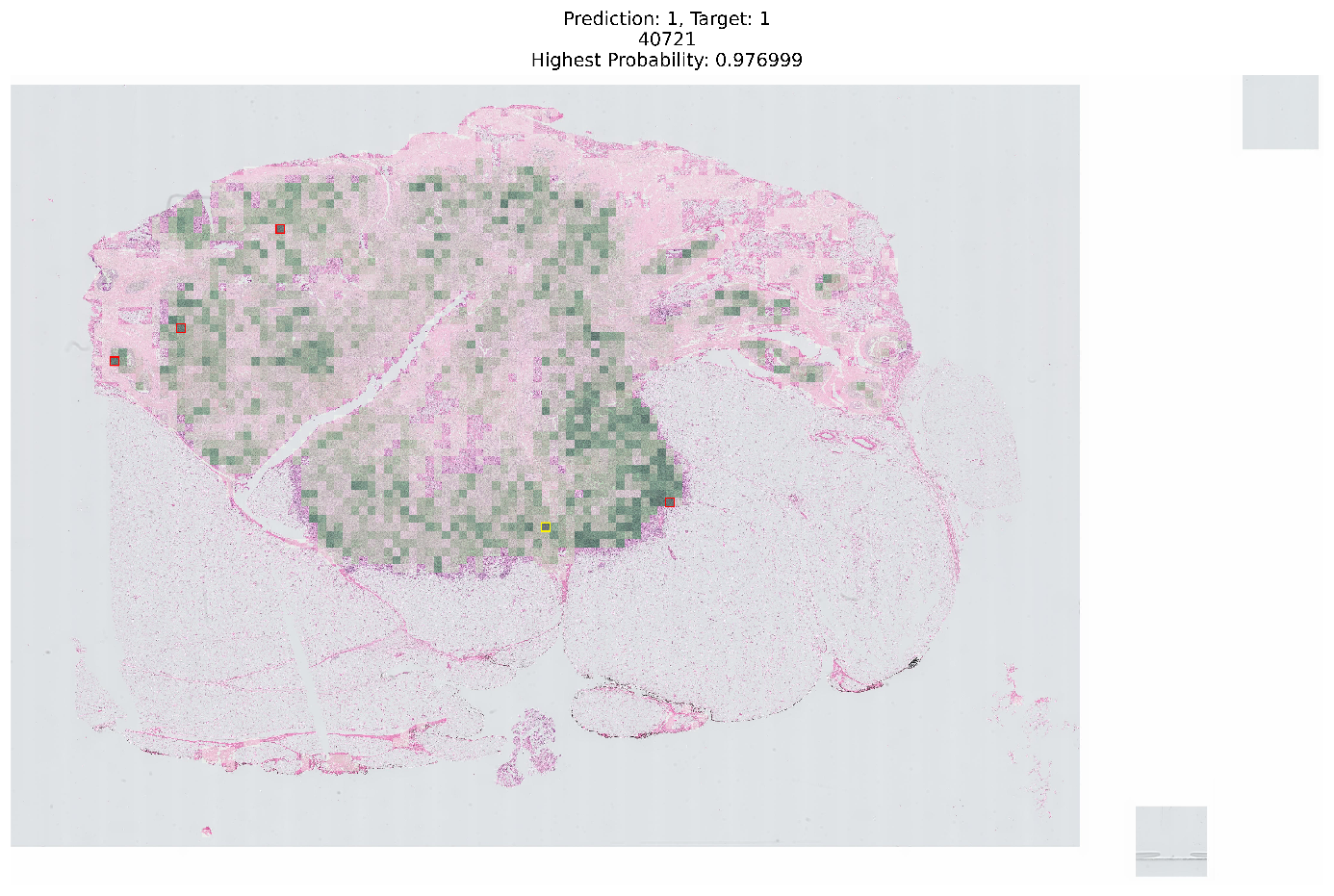


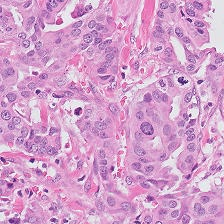

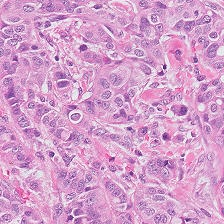

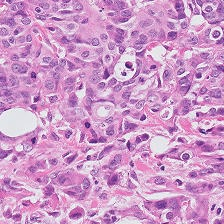

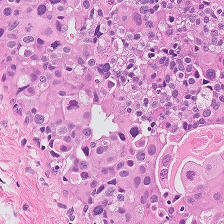

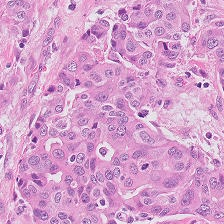


(g) Heatmap and top 5 tiles for a correctly classified high grade cancer.


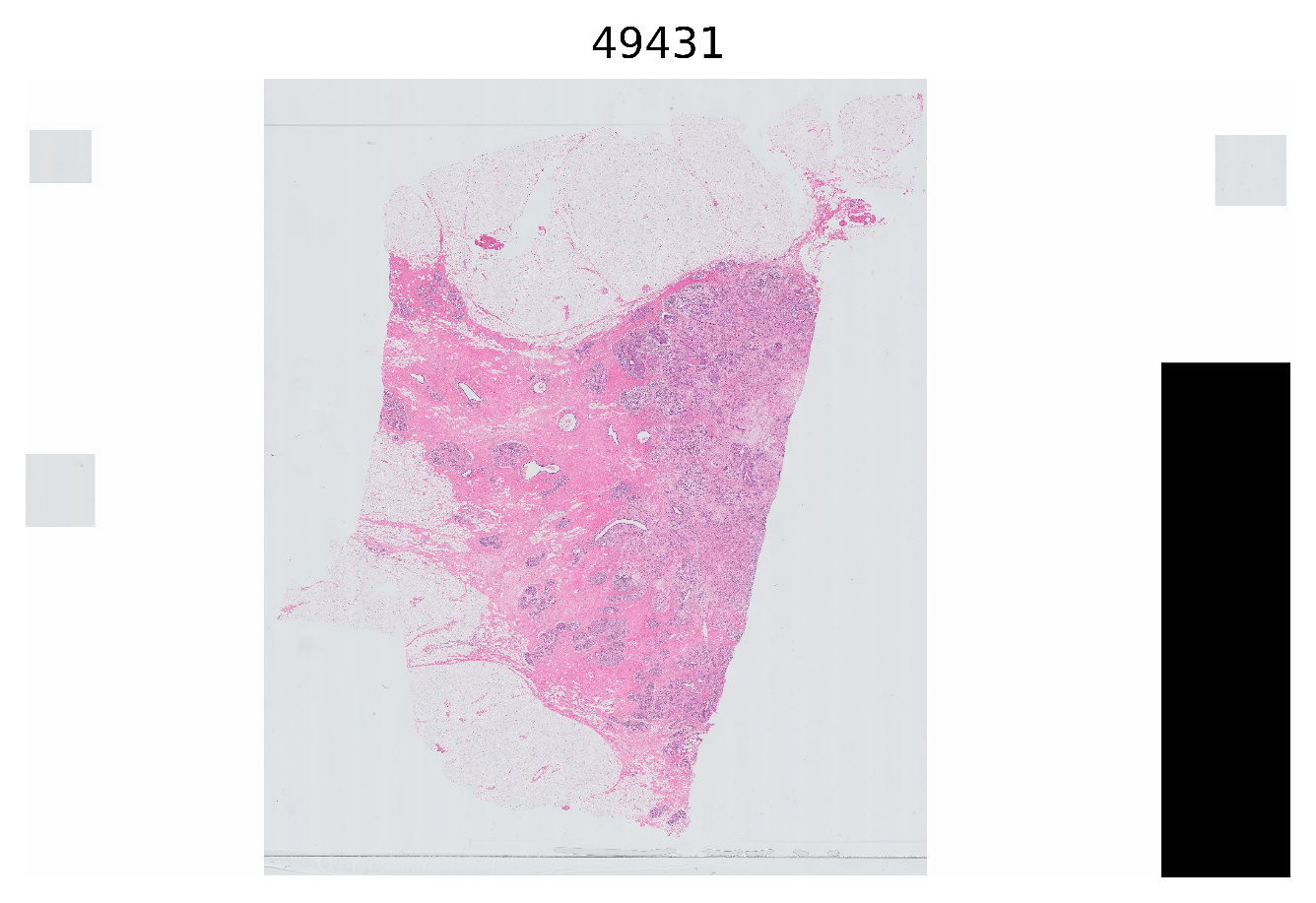

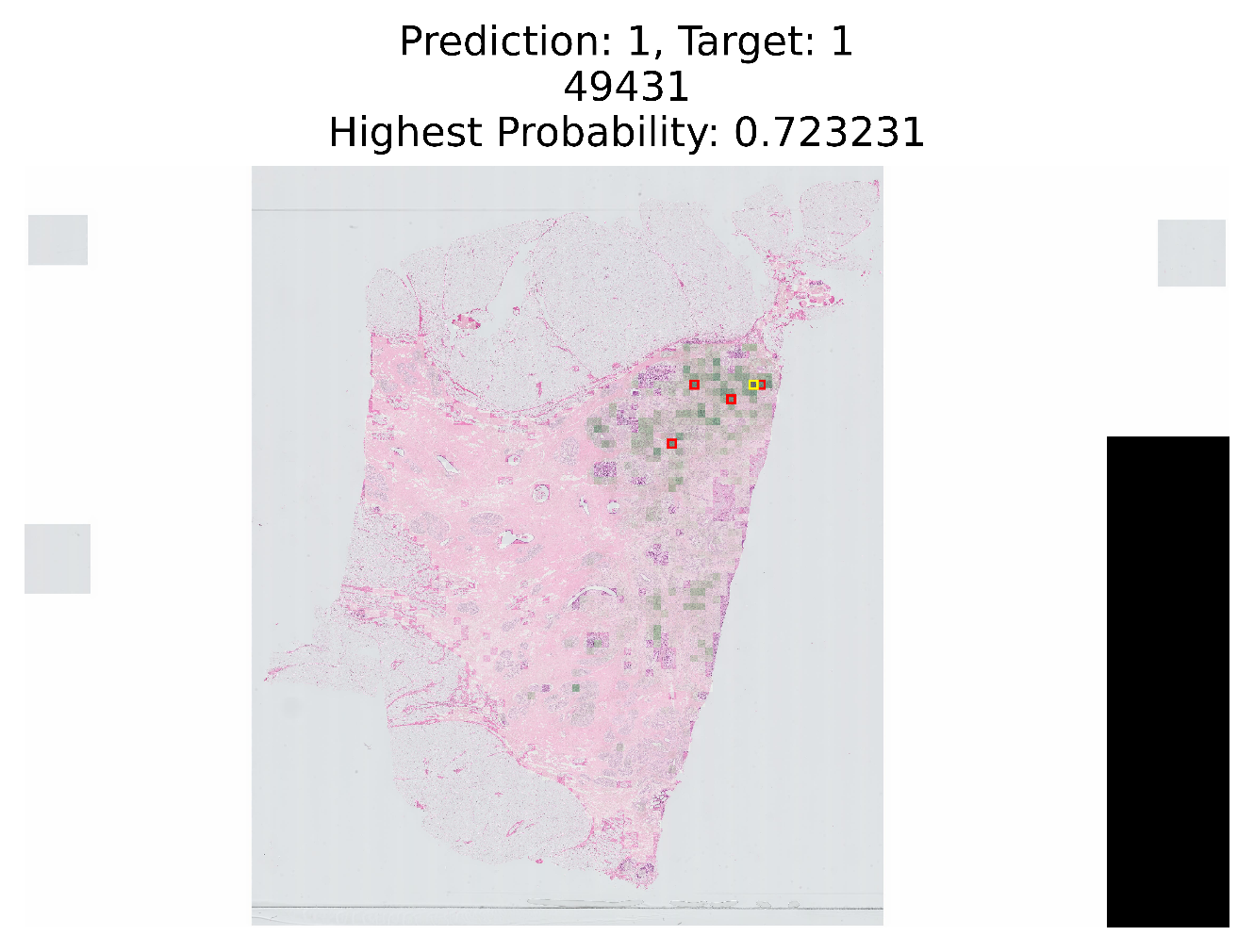


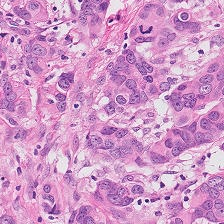

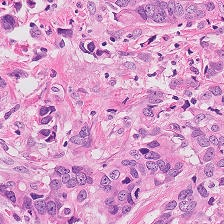

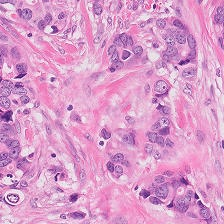

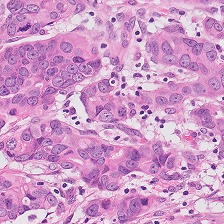

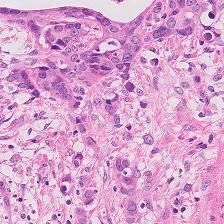


(h) Heatmap and top 5 tiles for a correctly classified high grade cancer.

**Figure S1**: Original WSI and WSI with heatmaps overlayed. The heatmaps are generated from the individual tile probabilities and run from white to dark green with white indicating low probability of high grade cancer and dark green indicating high probability of high grade cancer. The top 5 tiles with the highest probability are depicted with a red contour, and the tile with the highest probability is depicted with a yellow contour. The top 5 tiles are also shown at the bottom of each case. We show heatmaps for correctly predicted low-intermediate grade cancer (true negatives) (**a-b**), low-intermediate grade cancer predicted to be high grade (false positives) (**c-d**), high grade cancer predicted to be low-intermediate grade cancer (false negatives) (**e-f**) and correctly predicted high grade cancer (true positives) (**g-h**).

**Table S1:** Distribution of the tumor grades over the two different scanners used in the development and test datasets.

| Scanner grade distribution | Development dataset | Test dataset | P-value |
| --- | --- | --- | --- |
| Philips UFS scanner 1.6.1.3 RA (*n*( %)) |  |  | 0.12 |
| Grade 1 | 17 (19) | 13 (12) |  |
| Grade 2 | 36 (40) | 35 (33) |  |
| Grade 3 | 36 (40) | 58 (55) |  |
| Nanozoomer XR C12000-21/-22 (*n*( %)) |  |  | 0.48 |
| Grade 1 | 96 (16) | 76 (13) |  |
| Grade 2 | 208 (34) | 203 (35) |  |
| Grade 3 | 313 (51) | 301 (52) |  |
